# Long-term Observation of Regenerated Periodontium Induced by FGF-2 in the Beagle Dog 2-Wall Periodontal Defect Model

**DOI:** 10.1371/journal.pone.0158485

**Published:** 2016-07-08

**Authors:** Jun Anzai, Toshie Nagayasu-Tanaka, Akio Terashima, Taiji Asano, Satoru Yamada, Takenori Nozaki, Masahiro Kitamura, Shinya Murakami

**Affiliations:** 1 Pharmacology Department, Drug Research Center, Kaken Pharmaceutical Co., LTD, Yamashina-ku, Kyoto, Japan; 2 Department of Periodontology, Osaka University Graduate School of Dentistry, Suita, Osaka, Japan; Tokyo Medical and Dental University, JAPAN

## Abstract

The long-term stability and qualitative characteristics of periodontium regenerated by FGF-2 treatment were compared with normal physiological healing tissue controls in a Beagle dog 2-wall periodontal defect model 13 months after treatment by assessing tissue histology and three-dimensional microstructure using micro-computed tomography (μCT). After FGF-2 (0.3%) or vehicle treatment at the defect sites, serial changes in the bone mineral content (BMC) were observed using periodic X-ray imaging. Tissues were harvested at 13 months, evaluated histomorphometrically, and the cortical bone volume and trabecular bone structure of the newly formed bone were analyzed using μCT. FGF-2 significantly increased the BMC of the defect area at 2 months compared with that of the control group, and this difference was unchanged through 13 months. The cortical bone volume was significantly increased by FGF-2, but there was no difference between the groups in trabecular bone structure. Bone maturation was occurring in both groups because of the lower cortical volume and denser trabecular bone than what is found in intact bone. FGF-2 also increased the area of newly formed bone as assessed histomorphometrically, but the ratios of trabecular bone in the defect area were similar between the control and FGF-2 groups. These results suggest that FGF-2 stimulates neogenesis of alveolar bone that is of similar quality to that of the control group. The lengths of the regenerated periodontal ligament and cementum, measured as the distance from the defect bottom to the apical end of the gingival epithelium, and height and area of the newly formed bone in the FGF-2 group were larger than those in the control group. The present study demonstrated that, within the limitation of artificial periodontal defect model, the periodontal tissue regenerated by FGF-2 was maintained for 13 months after treatment and was qualitatively equivalent to that generated through the physiological healing process.

## Introduction

Exacerbation of periodontitis results in the destruction of periodontal tissue, which is difficult to regenerate using conventional periodontal treatments. To overcome this difficulty, guided tissue regeneration (GTR) was developed in the early 1980s, which uses a barrier membrane to keep the gingiva out of the defect, reserving space for periodontal regeneration with new bone, periodontal ligament (PDL), and cementum [1]. Although it is well-known that regenerated tissues obtained through GTR therapy can be maintained for a long-term basis [2–4], the results vary markedly depending on the dexterity and proficiency of the surgeon [5]. Furthermore, the installed barrier membrane may be a risk factor for infection [6]. Alternative treatment methods using autogenous, allogeneic, xenogeneic, and artificial bone grafts have also been developed. Moreover, the combination of 0.03% platelet-derived growth factor-BB (PDGF-BB) with granular β-tricalcium phosphate (β-TCP) as an artificial bone was approved by the US Food and Drug Administration in 2005 and is available for periodontal regeneration in the United States. These bone grafts are useful for achieving periodontal regeneration through osteoconduction and osteoinduction, but have limitations, such as their slow replacement with new bone, donor site morbidity by harvesting of autogenous bone, pathogen contamination, and anatomical limits on the amount of bone that can be harvested. Another treatment that is also applied clinically is the administration of an enamel matrix derivative extracted from porcine fetal teeth, Emdogain^®^, into the periodontal pockets. However, introduction of unknown pathogens from this animal product remains a concern.

To overcome these limitations and improve the success rate of periodontal regenerative therapy, we have been intensively investigating alternative methods using fibroblast growth factor-2 (FGF-2), which has been reported to enhance periodontal regeneration by activating the proliferation of mesenchymal progenitor cells in the periodontal tissue [7]. We previously demonstrated that FGF-2 enhances the regeneration of alveolar bone, cementum, and PDL in 2- and 3-wall defects [8], class II furcation defects [9], and 1-wall defects [10] in Beagle dog models and non-human primate class II furcation defect models [11]. We have also shown that 0.3% FGF-2 exhibited the maximal efficacy in inducing periodontal regeneration in a phase II clinical trial [12], and its effect was superior to an enamel matrix derivative in a phase III clinical trial [13]. However, the long-term stability and qualitative characteristics of the periodontium regenerated under FGF-2 treatment have not been compared with those created using the physiological healing process [14–16].

In the present study, we investigated the long-term stability and qualitative equivalence of the periodontal tissues regenerated 13 months after FGF-2 treatment by analysis of histology and three-dimensional micro-computed tomography (μCT) in a Beagle dog 2-wall periodontal defect model.

## Materials and Methods

### Animals

The highest standards of animal welfare were followed to respect the lives of the laboratory animals and to minimize their distress. Further, the study was conducted rationally using the minimum number of animals. Twenty-four female Beagle dogs (32–57 months of age) obtained from Kitayama Labes Co., Ltd (TOYO Beagle; Nagano, Japan) and Narc Co., Ltd (Nosan Beagle, Chiba, Japan) were used in this study. The animals were housed individually in stainless-steel cages in an animal room with controlled temperature at 18–26°C, relative humidity of 30%–70%, and 12-h light-dark cycle (light from 07:00 to 19:00). The animal room and cages were cleaned daily. During cage cleaning, the dogs were removed from the cages to reduce their stress. Water was available *ad libitum*, and 230 g of pelleted laboratory diet chow was provided daily. For 1 week after the surgery, a diet of soft dog food was provided. There was no animal became ill or died prior to the experimental endpoint. This study protocol was approved by the Animal Experiments Ethics Committee of Kaken Pharmaceuticals Co., Ltd (Authorization Number: S06-120, (Date of approval: Oct 28, 2006)).

The in-house regulations at Kaken comply with Japan’s Act on the Welfare and Management of Animals and the related international and domestic guidelines were followed. The ethics committee establishes, based on a review of the in-house regulations, whether all animal experimental protocols are written based on the “3Rs (Replacement, Reduction and Refinement) principles” before an experiment may begin and implements self-inspections and assessments of the animal experiment processes and the operations facility. Additionally, Kaken has been certified as a qualified institution for Laboratory Animal Care and Use by The Japan Health Sciences Foundation (Tokyo, Japan), which assesses and verifies compliance with the ‘Basic Guidelines for Proper Conduct of Animal Testing and Related Activities in the Research Institutions under the jurisdiction of the Ministry of Health, Labour and Welfare (Japan)’ as a third-party.

### Preparation of Test Substances

FGF-2 solution was prepared by dissolving 3 mg of freeze-dried FGF-2 in 1 mL of 3% hydroxypropyl cellulose (HPC) solution. For the negative control, 3% HPC solution itself was used.

### Tooth extraction

In order to make the region for defect creation, 4^th^ premolars were extracted. Beagle dogs were administered 2% Xylazine (Selactar, Bayer Yakuhin Ltd., Osaka, Japan, 1 mL/ body, intramuscularly) and pentobarbital (Nacalai tesque, Kyoto, Japan, 30 mg/kg, intravenously) for general anesthesia and 2% lidocaine with 0.00125% adrenaline (Xylocaine, Dentsply-Sankin K.K., Tokyo, Japan, 0.9 mL/site, intragingivally). Under anesthesia, the 4^th^ premolars were split through the furcation into two mesiodistal portions and removed using standard methods.

### Group assignment

After the wounds caused by the tooth extraction healed, 24 Beagle dogs were assigned to 3 groups of 8 animals balanced for BMC in this region. BMC was obtained by X-ray image analysis as follows.

Under general anesthesia, radiographic images of the defect sites were taken in the buccolingual direction with Dental X-ray film (BW-100, ISO size 2, Hanshin Technical Laboratory, Ltd. Hyogo, Japan) on an X-ray apparatus (CMBW-2; Softex Co., Ltd., Tokyo, Japan). The X-ray images were digitized, and then the bone mineral contents (BMC) of the defect sites were evaluated using an aluminum step wedge as a density standard and Simple PCI image analysis software (Compix Inc. Sewickley, PA, US).

### Periodontal Surgery and Administration of Test Substances

The Beagle dogs were anesthetized again as tooth extraction, a mucoperiosteal flap was raised, and 2-wall periodontal defects (mesiodistal width: 5 mm x depth: 4 mm; buccolingual width: 3 mm) were surgically created on the mesiobuccal portion of both sides of the mandibular 1^st^ molar. Alveolar bone was removed with a steel bar and the root surfaces were planed with Gracey curettes to remove the cementum and PDL. A notch was created on the root surface at the bottom of the bone defect, and the inner surface of the flaps were electrocauterized to prevent repair by the periosteum. Each of the eight dogs per group (16 total defects per group) received 60 μL of the HPC or FGF-2 solution in both mandibular defects. Eight dogs whose 4^th^ premolars were extracted were also used without creating defects as intact periodontal tissue samples in this study. The gingival flaps were sutured closed, and penicillin (4000 units/dog) and streptomycin (200 mg/dog) were administered subcutaneously to prevent infection after surgery. Treated sites were observed more than two times per week until wound healed and antibiotics were injected. Dental calculus was removed at the time of taking the X-ray photographs as oral care after post-surgery.

### Measurement of BMC in the defect site

Radiographic imaging of the defect sites was performed before and at 0 and 2 weeks, and 1, 2, 3, 5, 7, 9, 11, and 13 months after defect creation. BMC in the defect site was measured as described above. BMC data for the defect is shown in increments from the time of defect creation. The BMCs are reported as the equivalent thickness of aluminum (mm Al eq.). The study endpoint (13 months after administration) was determined as the time when there was no statistical difference between the BMCs measured for three consecutive time points in both the control and FGF-2 groups.

### Preparation of Tissue Specimens

Under general anesthesia, the Beagle dogs were euthanized by exsanguination at 13 months after the FGF-2 or vehicle administration, and the tissues containing the defect region were harvested and fixed with neutral-buffered 10% formalin.

### Evaluation of the Microstructure of the Newly Formed Bone in the Defect Site

**Fig 1:**
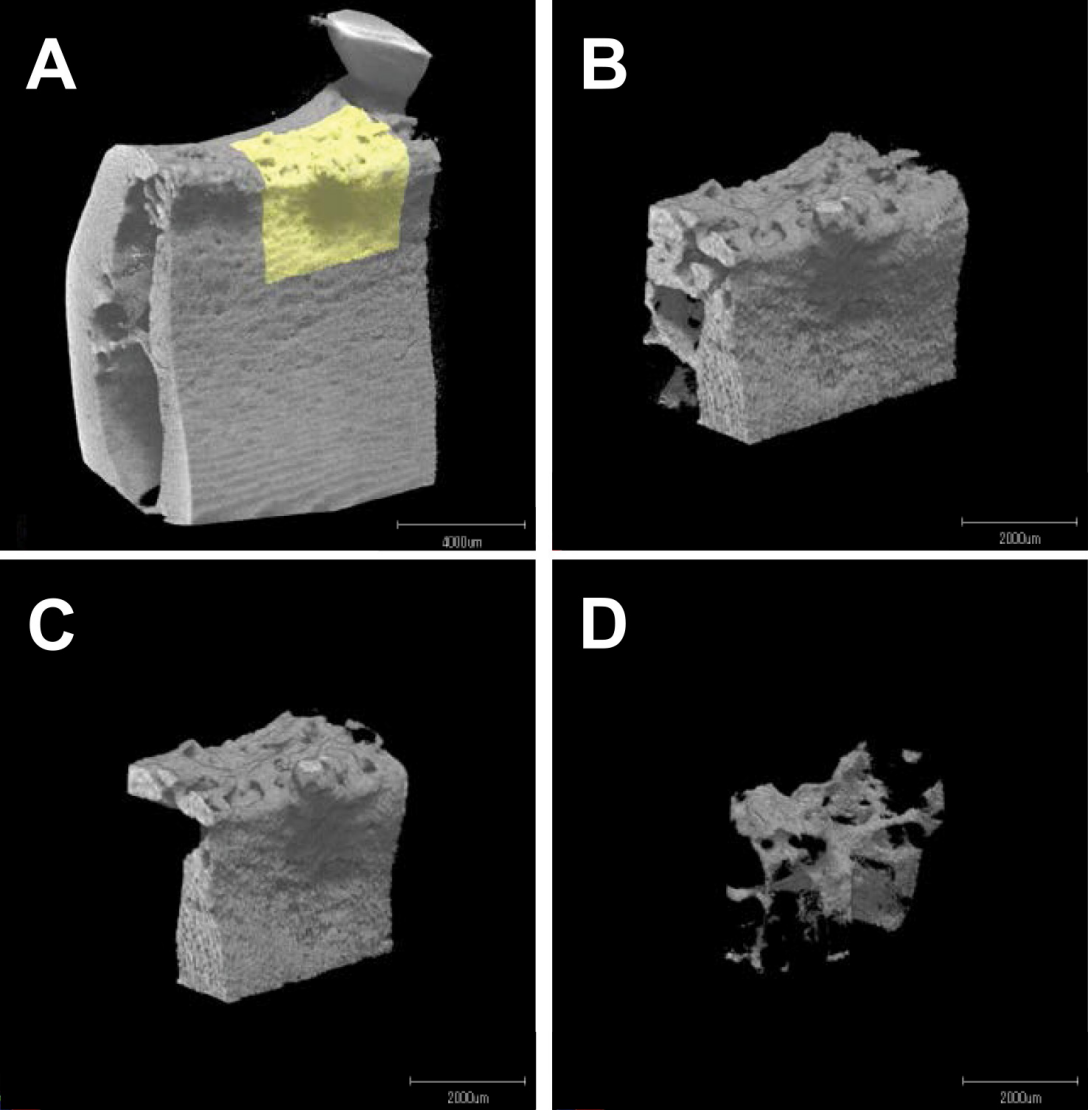
μCT images of the defect site. **A**, Region where the defect was created (yellow) in the surrounding periodontal tissue. **B**, Newly formed bone in the defect region (yellow region in A). **C**, The cortical bone in B. **D**, The trabecular bone in B.

The μCT images of the newly formed bone in the defect sites (Fig 1A and 1B) were obtained using micro focus X-ray CT (ScanXmate-E090S40 in vivo; Comscantecno Co., Ltd., Yokohama, Japan) and analyzed by TRI/3D-BON (Ratoc System Engineering, Tokyo, Japan). The defect region (mesiodistal width: 5 mm x depth: 4 mm; buccolingual width: 3 mm) in the alveolar bone was defined based on the notch on the root surface in the computer software. The corresponding site in the intact group was defined based on the alveolar crest. The lingual and medial 0.5 mm of tissue into the defect site were removed from the analysis because these areas were indistinguishable from the existing bone. Additionally, the distal 1 mm volume of tissue was also removed because this area had a different bone structure because it was facing the tooth. The corresponding sites in the intact group were analyzed as an intact bone structure control (n = 16). In this analysis, after the cortical and trabecular bones were virtually separated (Fig 1C and 1D), the cortical bone volume (CBV), tissue volume (bone marrow volume; TV), %bone fill ((CBV+TV) × 100/mean intact (CBV+TV)) bone volume/tissue volume (BV/TV), trabecular thickness (Tb.Th.), trabecular separation (Tb.Sp.), and trabecular number (Tb.N.) were calculated.

### Histology and Histomorphometry

After μCT scanning, the tissues were decalcified for 2 months in 10% formic acid before being embedded in paraffin using standard methods. Next, serial sections were cut from the lingual to buccal direction in the defect site, and the sections 1200 μm from the lingual side of alveolar bone were stained with Azan. Four defects (two from each group) were excluded from this study because of remaining cementum, caused by insufficient root planing, and for tooth abrasion and alveolar bone resorption caused by abnormal occlusion.

Histometric measurements were made on these specimens using WinRoof image analysis software (ver.5.03, Mitani-Sangyo, Tokyo, Japan). Apical extension of the notch was used to mark the reference points. The following six histometric parameters were calculated [11,17,18]. 1) The area of newly formed bone in the defect (ANB); 2) the ratio of trabecular bone matrix area in the ANB (RTB); 3) the height of the newly formed bone defined as the distance from the notch to the coronal extension of the newly formed bone along the root surface (HNB); 4) the length of the regenerated PDL defined as the distance from the notch to the coronal extension of the newly formed PDL (LPL); 5) the length of the regenerated cementum defined as the distance from the notch to the coronal extension of the newly formed cementum (LCM); and 6) the distance to the junctional epithelium defined as the distance from the notch to the apical extension of the junctional epithelium (DJE).

The observations of the cementum and PDL were scored according to the following criteria. For the cementum: very thin (1); thinner than the intact cementum, but of a constant thickness (2); or of a similar thickness as the intact cementum (3). For the PDL: narrow collagen fibers that ran parallel to the root surface (1); narrow collagen fibers that ran parallel and obliquely to the root surface (2); thick collagen fibers that ran obliquely to the root surface (3).

### Statistical Analysis

All of the parameters are shown as the mean and standard deviation (S.D.). Differences in BMC between the control and FGF-2 groups were assessed with Student’s t-tests at each time point. The microstructure analysis parameters (CBV, TV, %bone fill, BV/TV, Tb.Th., Tb.N., and Tb.Sp.) were analyzed with Tukey’s multiple comparison tests. The histometric measurement parameters were compared with Student’s t-tests. These statistical analyses were performed with SPSS statistical software (ver.14.0.2., SPSS Inc. Chicago, IL), and p-values less than 0.05 were considered statistically significant.

## Results

### Change of BMC in the Mandibular Defect Region

**Fig 2:**
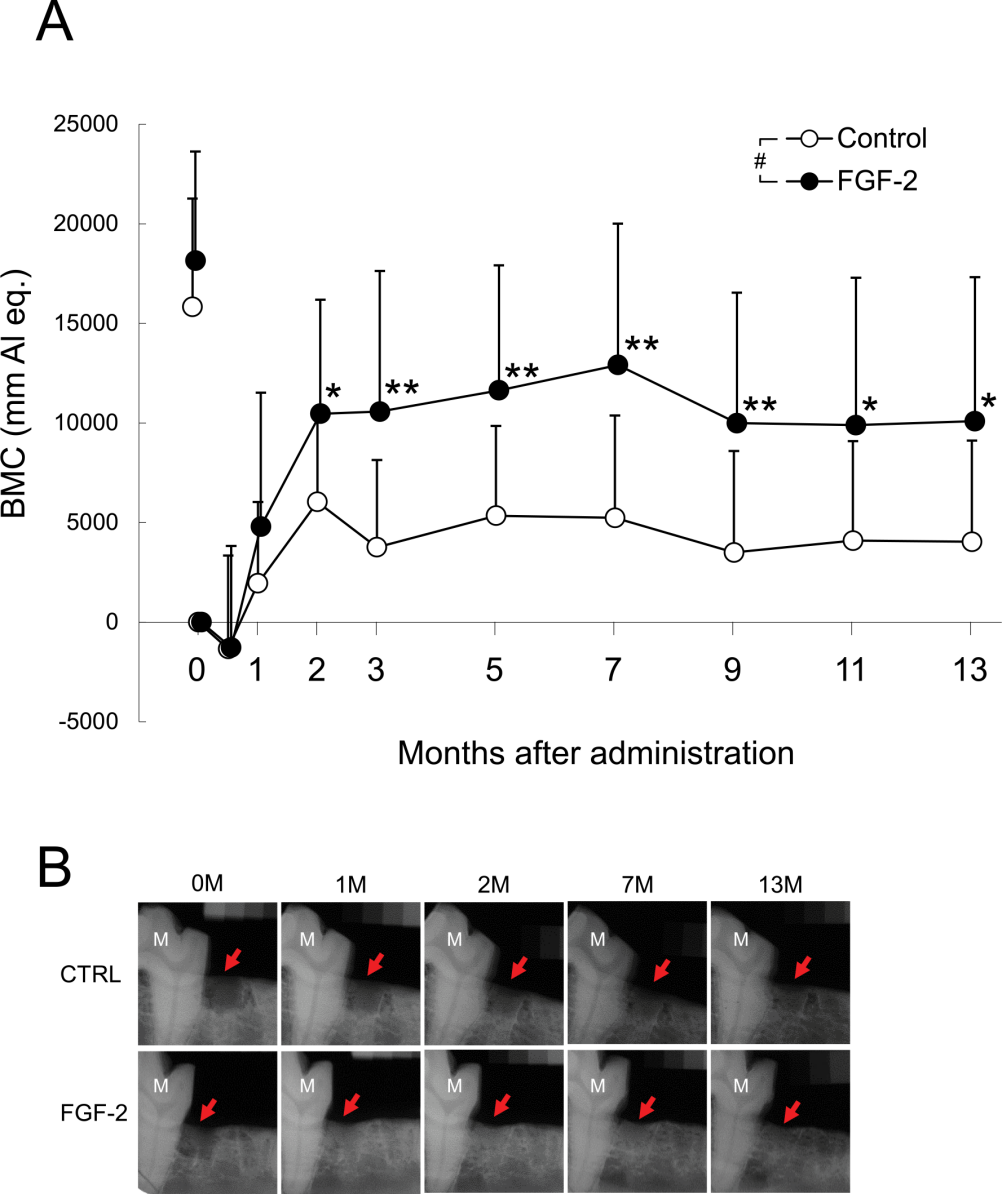
Chronological change of newly formed bone in the defect region. **A**: BMC. Mean±S.D., n = 14, *: p < 0.05, **: p < 0.01(vs control, Student’s t-test). #: p < 0.05 (vs control, repeated measurement ANOVA). Open circle and black circle showed the values of the control, and the FGF-2 groups, respectively. The symbols of left side showed the value before defects creation. **B**: X-ray images. X-ray images of 0, 1, 2, 7, and 13 months were shown. M: 1st molar. Red arrow: defect region. Upper column: Control, Lower column: FGF-2.

The BMC of the defect region in the control group increased within 2 months after defect creation and remained unchanged through 13 months (Fig 2A and 2B). The BMC of the FGF-2 group also increased within 2 months, but was significantly higher than that of the control group from 2 to 13 months. In both groups, the BMC level plateaued from 9 to 13 months (control: p = 0.738; FGF-2: p = 0.903; repeated measures ANOVA of three consecutive time points). Thus, 13 months was established as the study endpoint. This result suggested that the bone metabolism stabilized at 9 months after defect creation.

### Microstructure Analysis of the Newly Formed Bone

**Fig 3:**
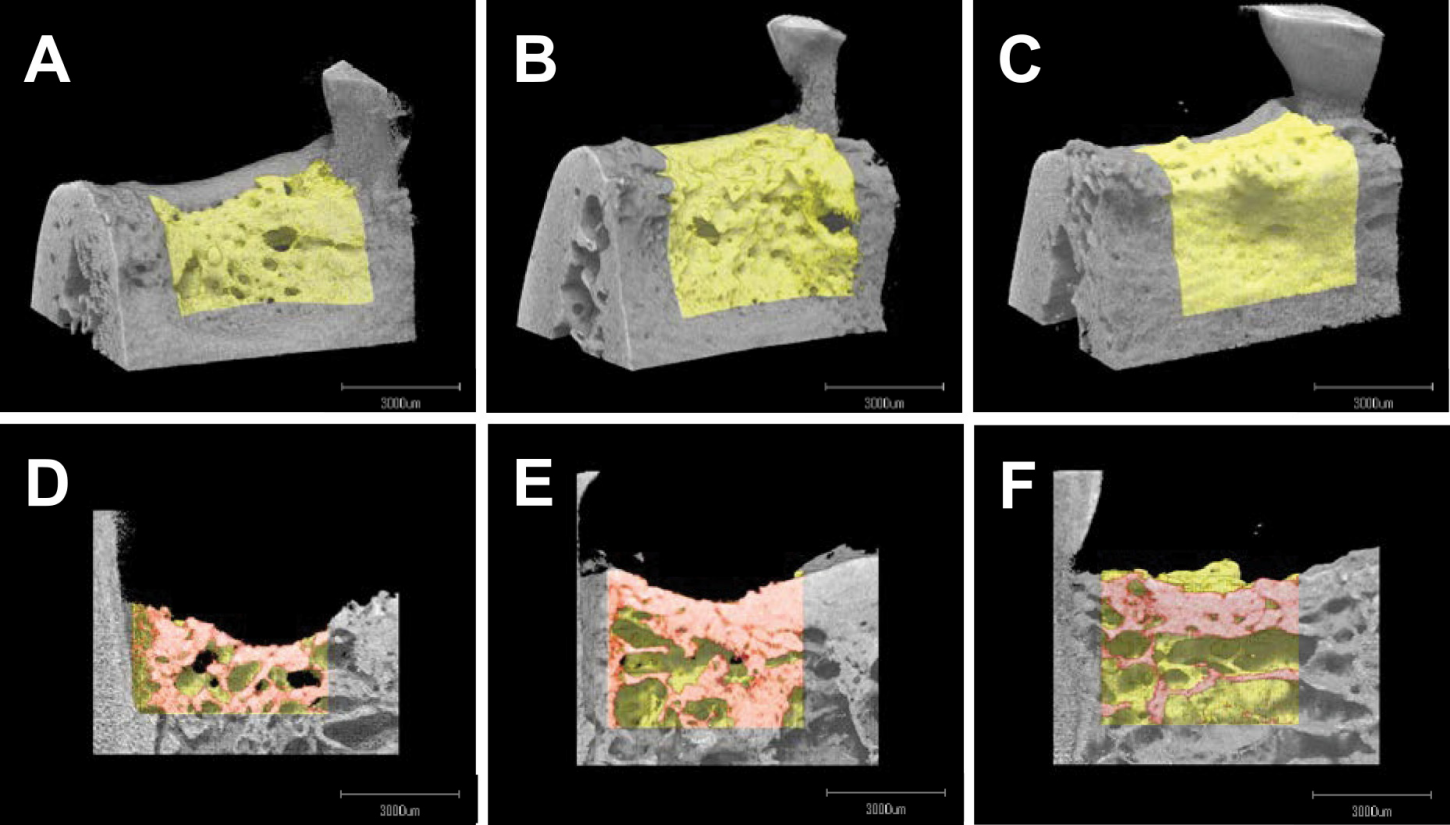
Representative μCT images. Upper column: buccal images of the defect region (newly formed bone shown in yellow). Lower column: sectional images of the defect region (red) observed from the lingual site. **A**, **D**: control; **B**, **E**: FGF-2; and **C**, **F**: intact groups.

The volume and microstructure of the newly formed bone in the defects at 13 months was analyzed using X-ray images acquired by μCT to evaluate bone quality (Fig 3).

**Fig 4:**
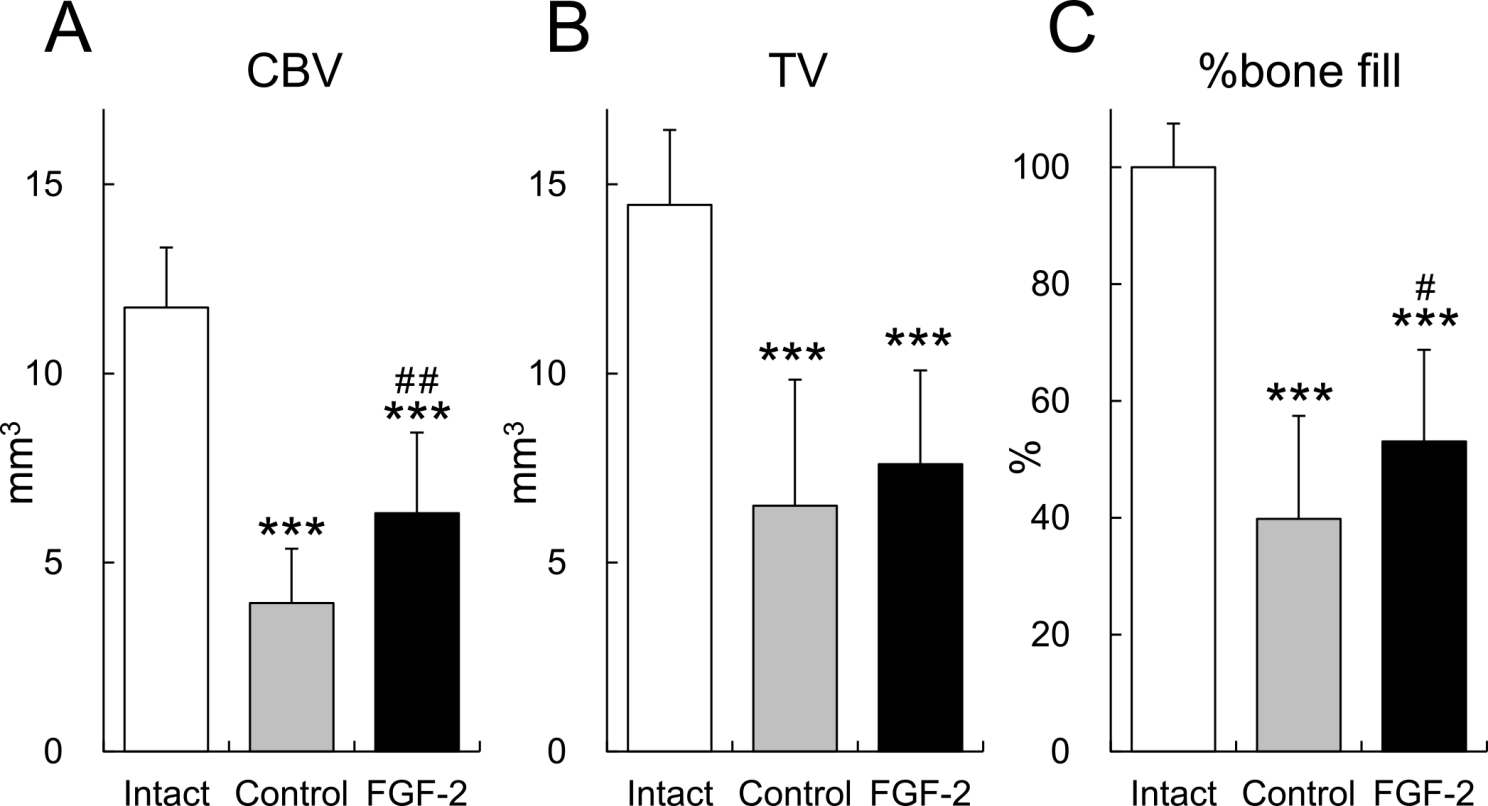
Cortical bone volume in the defect region. **A**: cortical bone volume, **B**: tissue volume (bone marrow volume), and **C**: %bone fill. Mean±S.D., n = 14 (control and FGF-2) or 16 (intact), ***: p < 0.001 (vs intact, Tukey’s multiple comparison test), #: p < 0.05, ##: p < 0.01 (vs control, Tukey’s multiple comparison test).

The CBV and %bone fill of the FGF-2 group were significantly higher than that of the control group (p = 0.002 and p = 0.042, respectively; Fig 4). There was no difference in TV between the control group and the FGF-2 group. The CBV, TV, and %bone fill of the control group and the FGF-2 group were low compared with those of the intact group.

**Fig 5:**
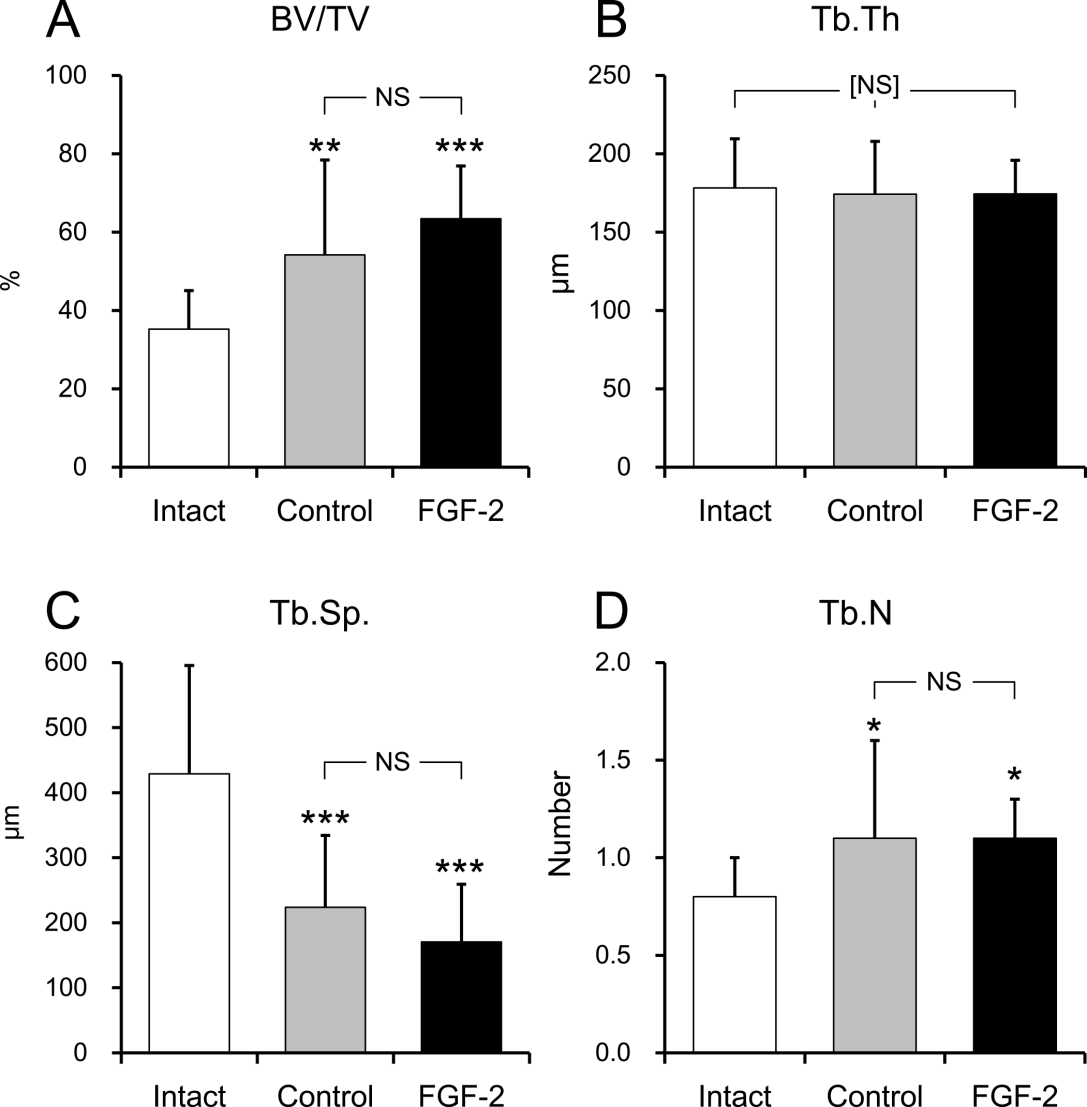
Microstructure analysis of the newly formed bone. **A**, Bone volume/tissue volume (BV/TV); **B**, trabecular thickness (Tb.Th.); **C**, trabecular separation (Tb.Sp.), and **D**, trabecular number (Tb.N). Mean ± S.D. of n = 14–16. NS: not significant vs. the control group, Tukey’s multiple comparison test. [NS]: not significant between any pairs of groups, Tukey’s multiple comparison test. *: p < 0.05, **: p < 0.01, and ***: p <0.001 vs. the intact group, Tukey’s multiple comparison test.

In the trabecular bone, there were no differences in BV/TV, Tb.Th., Tb.Sp., or Tb.N. between the control and FGF-2 groups (p = 0.325; Fig 5A–5D). These results suggested that the microstructure of the newly formed bone in the FGF-2 group had similar properties as the bone regenerated in the control group. However, compared with the intact group, the BV/TVs and Tb.N.s of the control and FGF-2 groups were high (BV/TV; p = 0.009, p < 0.001, respectively; Fig 5A, Tb.N; p = 0.020, p = 0.026, respectively; Fig 5D) and Tb.Sp. was low (p < 0.001 for both groups; Fig 5C). There was no difference in Tb.Th. (Fig 5D) among any of the groups. These results suggested that the newly formed bone was a more trabeculae-rich and denser structure than intact bone, regardless of FGF-2 treatment.

### Histology and Histomorphometry

**Fig 6:**
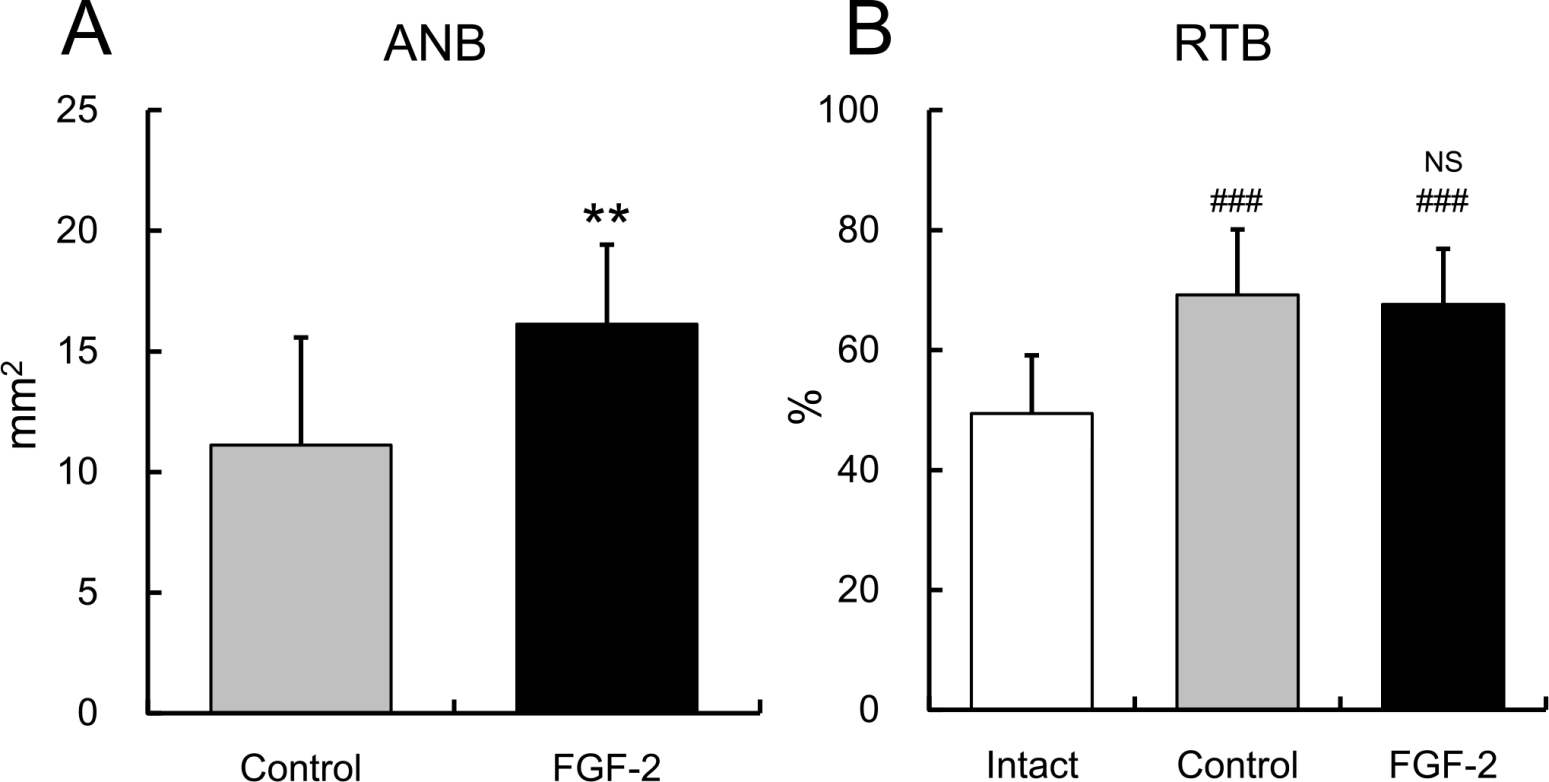
Histomorphometric analysis of the area of newly formed bone and the ratio of trabecular bone. **A**: area of the newly formed bone (ANB), and **B**: ratio of the trabecular bone (RTB). Mean ± S.D. of n = 14–16. NS: not significant vs. the control group, Tukey’s multiple comparison test. **: p < 0.01 vs. the control group, Student’s t-test. ###: p < 0.001 vs. the intact group, Tukey’s multiple comparison test.

ANB was significantly higher in the FGF-2 group than in the control group (p = 0.002; Fig 6A), whereas the RTBs of the both groups were not different from each other (p = 0.895), but were higher than that of the intact group (p < 0.001 for both groups; Fig 6B). Therefore, the newly formed bone in the defect of the FGF-2 group had a similar bone density as that in the control group.

**Fig 7:**
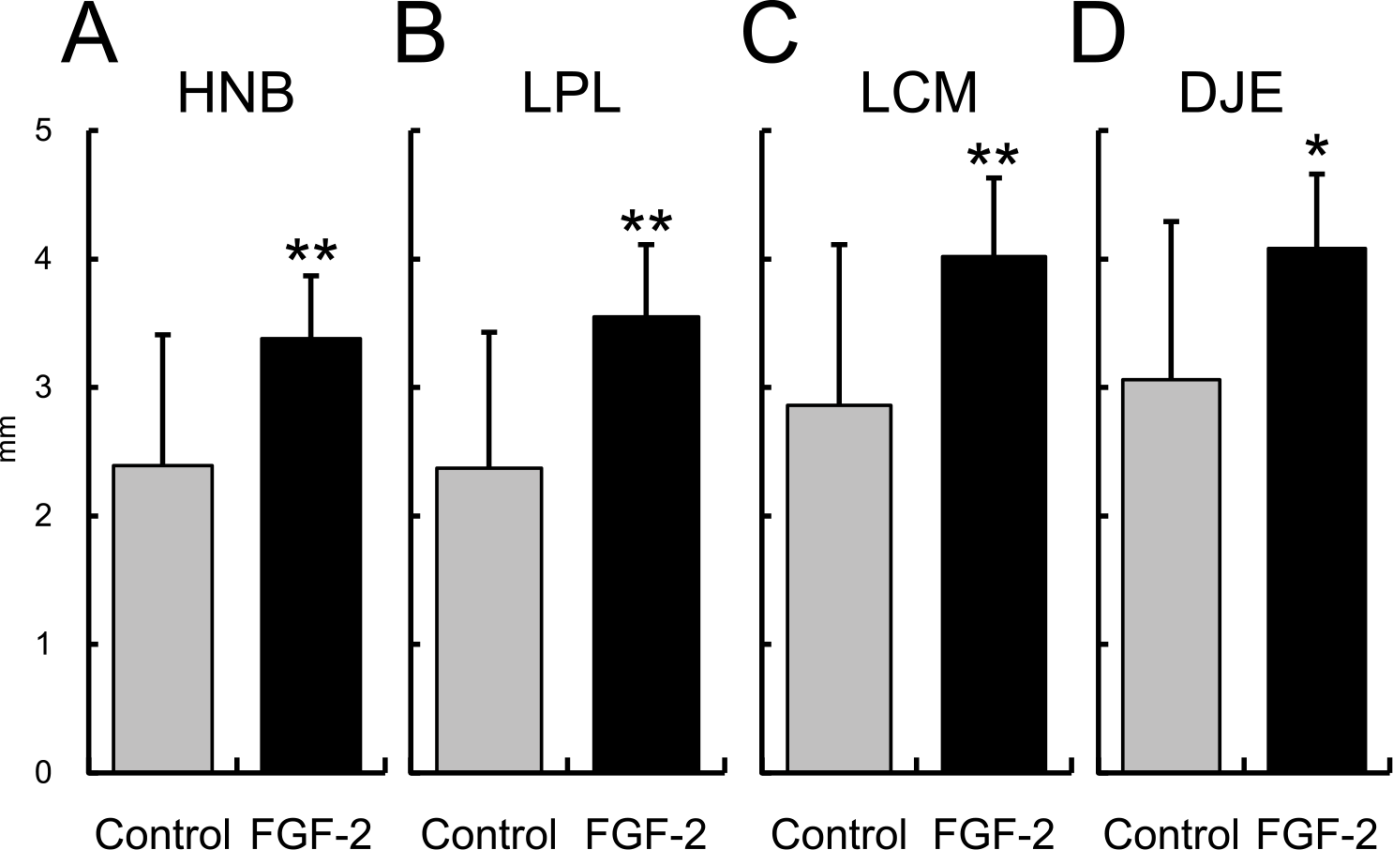
Histomorphometric analysis of the connective tissue attachment. **A**: height of the newly formed bone, **B**: length of the regenerated PDL, **C**: length of the regenerated cementum, and **D**: distance to the junctional epithelium. Mean ± S.D. of n = 14. *: p < 0.05 and **: p < 0.01 vs. the control group, Student’s t-test.

**Fig 8:**
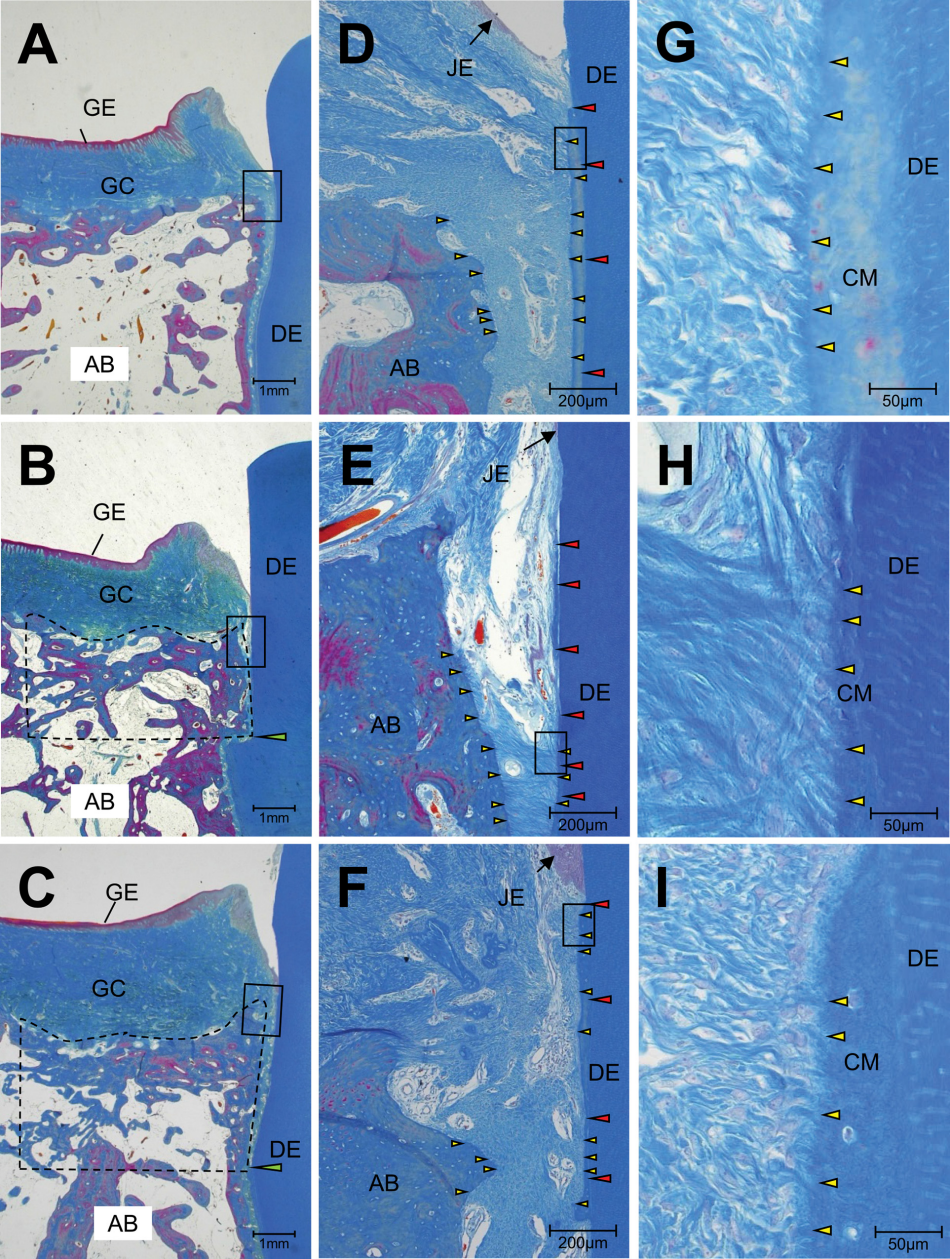
Representative images of newly formed bone and newly formed connective tissue attachment. Azan staining. **A**, **D**, and **G**; intact, **B**, **E**, and **H**; control, and **C**, **F**, and **I**; FGF-2. Regions bounded by dotted line showed the newly formed bone in the defect. Green arrow heads; the bottoms of the defects. Red arrow heads; cementum, yellow arrow heads; collagen fiber bundle. Magnified images corresponding to the box in A, B, C, D, E, and F were Shown in D, E, F, G, H, and I, respectively. GE: gingival epithelium, GC: gingival connective tissue, AB: alveolar bone, JE: junctional epithelium, DE: dentin, CM: cementum.

The HNB, LPL, LCM, and DJE of the FGF-2 group were significantly higher than those of the control group (p = 0.003, p = 0.001, p = 0.004, and p = 0.010, respectively; Fig 7A–7D). These results showed that FGF-2 enhanced the formation of new connective tissue attachment along the exposed root surface. The tissue specimens stained with Azan showed varied staining from dark blue to light purple in the newly formed bone (inside the dotted line) in the control, FGF-2, and intact groups (Fig 8), suggesting that natural bone matrices were regenerated. The newly formed cementums of both groups were thinner than the intact cementum (Table 1). The magnified region corresponding to the box indicated in Fig 8A, 8B, 8C, 8D, 8E and 8F are shown in Fig 8D, 8E, 8F, 8G, 8H and 8I, respectively. The histological observations of the regenerated PDL in both groups were similar to that found in the intact group. The newly formed PDL had an appropriate width from the bottom of the defect to the top of the newly formed bone, and the collagen fiber bundles inside the PDL were inserted into the cementum and bone.

**Table 1:** Histological findings of newly formed cementum and periodontal ligament.

Group	Cementum			Periodontal Ligament		
	Score			Score		
	1	2	3	1	2	3
Intact	0	0	16	0	0	16
Control	1	12	0	0	3	10
FGF-2	0	14	0	0	0	14

N = 13 (the control group), 14 (the FGF-2 group) and 16 (the intact group). One defect in the control grope was removed because of no regeneration of cementum and PDL.

Score: cementum. 1: very thin, 2: thinner than the intact cementum, but a constant thickness, and 3: the similar thickness to the intact cementum.

Score: periodontal ligament. 1: collagen fibers were narrow and ran parallel to the root surface, 2: collagen fibers were narrow and ran parallel and obliquely to the root surface, and 3: collagen fibers were thick and ran obliquely to the root surface.

Downgrowth of the junctional epithelium was observed in one defect in the control group, but was not observed in the FGF-2 group. Ankylosis was not seen in either group.

Taken together, these results indicate that FGF-2 enhanced the regeneration of the periodontal tissue with the same quality as that created by the normal physiological healing process with respect to bone density, matrices, and connective tissue attachment.

## Discussion

In the present study, we investigated the chronological changes in the BMC of Beagle dog periodontal defect sites and evaluated the quantity and quality of the regenerated periodontal tissue 13 months after treatment with FGF-2 or vehicle control. Consistent with previous studies that evaluated repair at 1 or 2 months [8,9,11], the newly formed bone area, cementum, and PDL of the FGF-2 group were larger than those of the control group. Therefore, the periodontium regenerated under FGF-2 treatment was suggested to be maintained for a long period of time. The microstructure of the newly formed bone induced by FGF-2 was equivalent to that formed by the normal physiological healing process observed in the control group. These results suggest that the newly formed bone in the control and FGF-2 groups were of similar quality. However, the newly formed bone was denser than intact alveolar bone regardless of treatment group. The morphology of the newly formed cementum and PDL were also similar in the control and FGF-2 groups.

Using radiographic imaging, the BMC of the FGF-2 group was found to be increased beginning 1 month after administration, and significant differences between the BMCs of the FGF-2 and control groups were observed from 2 months onward (Fig 2A). This finding is supported by the histological measurement of ANB (Fig 6A) and the μCT analysis of CBV (Fig 4A), indicating that this increase in BMC in the FGF-2 group represented an enhanced formation of new bone. Therefore, FGF-2 promoted the formation of new bone in the defect site within 2 months, and that newly formed bone was maintained through 13 months.

The increases in newly formed bone in the control and FGF-2 groups began at the same time point and the decreases between 7 months and 9 months regarded as remodeling also progressed in parallel in both groups (Fig 2A). These results suggest that FGF-2 enhanced the physiological bone formation. FGF-2 likely induces bone formation by promoting the proliferation of undifferentiated cells in the bone marrow [19,20], which then differentiate into osteoblasts. Additionally, STRO-1^+^/CD146^+^ cells with multi-differentiation potential were reported to exist in the PDL [21] and to proliferate in response to FGF-2 [22]. In fact, proliferating nuclear antigen-positive cells derived from bone marrow and PDL were reported to be markedly increased by FGF-2 at 7 days after administration in a Beagle dog 3-wall periodontal defect model [7]. Therefore, because FGF-2 was responsible for the proliferation of recruited pluripotent cells and these cells differentiated adapting to surrounding environment, new bone was formed physiologically.

Cortical bone volume of the FGF-2 group increased compared with that of the control group (Fig 4A). Mentioned above, FGF-2 stimulates bone marrow-derived stromal cells, and intravenous injection of FGF-2 induce new bone formation within bone marrow cavity, and result in thickening of cortical bone in normal and ovariectomized rats [19,23]. In the animal model in this study, bone regeneration occurred from bone marrow side and newly formed bone matured into cortical bone on the surface contacting with gingiva in our previous study (S1 Fig). Taken together, increase in cortical bone volume by FGF-2 was thought to be due to stimulation of the cells within bone marrow.

The TV and %bone fill of the control and the FGF-2 groups have small difference and were low compared with those of the intact group (Fig 4B and 4C). Bone in the flap side is more responsible for these volumetric analyses with μCT because of deletion of bone in the wall sides with vague border between newly formed bone and the existing bone. But, in a 2-wall periodontal defect model using in this study, regeneration of newly formed bone is high in wall side and low in flap side. For these restrictions, the differences among groups were not thought to fit completely to other analyses. An analysis that can compare the defect region between 0 and 13 months after defect creation may be needed for more accurate results.

The qualities of the newly formed bone, including the 3D structure of the trabecular bone, were assessed using μCT. The bone structure and mass are important factors affecting bone quality [24]. μCT analysis is a powerful tool for evaluating bone quality because it allows the 3D microarchitecture analysis of the bone [25]. Quantitative image processing of 3D bone structures is critical for assessing bone quality [26]. At 13 months, there were no differences between the control and FGF-2 groups in any of the parameters that describe the structure of the trabecular bone (Fig 5). Moreover, although the ANB of the FGF-2 group was significantly higher than that of the control group (Fig 6A), there was no difference in RTB as the 2D bone density (Fig 6B) between those groups. These results indicate that the quality of the newly formed bone induced by FGF-2 was similar to that created by the normal physiological healing process.

However, compared with the intact mandibular bone, the RTBs (Fig 6B) and BV/TVs (Fig 5) of both the control and FGF-2 groups were significantly higher. Moreover, the Tb.Sp. were lower, while the Tb.N. was higher, in the control and FGF-2 groups (Fig 5) than the intact bone. Taken together, the trabecular bone structures of these groups were denser than that of the intact mandibular bone. In general, newly formed bone is absorbed through bone remodeling, converted to mature bone, and eventually achieves the same structure and quality as normal bone. Although the bone metabolism appeared to have completed by 13 months because of the plateau in BMC, the newly formed bone was likely still in the process of maturation, and would be remodeled over time when exposed to mechanical stimuli such as mastication. Moreover, because CBVs of the control and the FGF-2 groups were lower than that of intact group, cortical bone formation was considered to be ongoing. Huja *et al*. showed that BV/TV and bone density decreased and pressure resistibility increased in the trabecular bone of the mandibular condyle with maturation induced by mastication by comparing young and old dogs [27]. Therefore, we believe that the trabecular bone structure and cortical bone thickness of the newly formed bone in both the control and FGF-2 groups would continue to gradually change over a long time, gradually approaching the structure of natural bone.

FGF-2 increased the lengths of the newly formed cementum and PDL (Fig 7B and 7C), resulting in the regeneration of the connective tissue attachment 13 months after defect creation. We previously reported that FGF-2 promoted the formation of a new cementum and PDL at an earlier time point, such as 6 or 8 weeks [9,28]. Compared with this result, the present study suggests that the connective tissue attachment regenerated by FGF-2 was also maintained for a long time, from formation at 2 months until at least 13 months.

The regenerated PDLs in the control and FGF-2 groups appeared to be as mature as that in the intact group, whereas the regenerated cementum was thinner than the intact cementum (Table 1). It is well-known that cementum, unlike bone, continues to thicken over time through successive accumulation [29], and regenerated cementum follows a similar process [30,31]. Lee *et al*. found that, after GDF-5 administration in a 1-wall Beagle dog periodontal defect model [32], the regenerated cementum was thicker at 24 weeks than at 8 weeks. These reports supports that the regenerated cementum in the present study thickened gradually over time.

The present study was performed with not a clinically-encountered chronic periodontal defect but a surgical created model of which the healing potential would be higher [33]. Under this condition, FGF-2 promoted an increase in alveolar bone, cementum, and PDL in a Beagle dog 2-wall periodontal defect model as well as in various animal models of periodontal diseases [8–11]. The present study clearly demonstrates that periodontal tissue regenerated by FGF-2 treatment was maintained through 13 months after administration, and was qualitatively similar to that created by the normal physiological healing process, which can be well integrated into the existing periodontal tissue.

## Supporting Information

S1 Fig Chronological change of newly formed bone—μCT images -. **A**: 1month, **B**: 3.5 months, **C**: 6 months, and **D**: 9 months after defect creation and FGF-2 administration. These μCT imeges were obtained from the previous study.
(EPS)
